# Supplementary material for: A dual-reporter system for investigating and optimizing protein translation and folding in E. coli
Source: Nat Commun. 2021 Oct 19;12:6093. doi: 10.1038/s41467-021-26337-1 (PMC8526717; doi:10.1038/s41467-021-26337-1)
Supplement: Supplementary file 1 — Supplementary information [file 41467_2021_26337_MOESM1_ESM.docx]

**­A dual-reporter system for investigating and optimizing protein translation and folding in *E. coli***

Ariane Zutz^1,3^, Louise Hamborg Nielsen^1,2^, Lasse Ebdrup Pedersen^1^, Maher M. Kassem^2^, Elena Papaleo^2^, Anna Koza^1^, Markus Herrgard^1^, Sheila Ingemann Jensen^1^, Kaare Teilum^2^, Kresten Lindorff-Larsen^2^, Alex Toftgaard Nielsen^1#^

^1^The Novo Nordisk Foundation Center for Biosustainability, Technical University of Denmark, Kemitorvet, 2800 Kgs. Lyngby, Denmark

^2^Structural Biology and NMR Laboratory, Department of Biology, University of Copenhagen, Ole Maaloes Vej 5, 2200 Copenhagen N, Denmark

^#^Corresponding author

Phone: +45 45258010

Postal address: Building 220, Kemitorvet, 2800 Kgs. Lyngby, Denmark

Email: [atn@biosustain.dtu.dk](mailto:atn@biosustain.dtu.dk)

Supplementary Table 1. Primer sequences

| Primer name | Primer sequence 5’- 3’ |
| --- | --- |
| *GFP-fwd* | AGTATCTAGAATGCGTAAAGGAGAAGAACTT |
| *GFP-ASV-rev* | ACTGACTAGTTTAAACTGATGCAGCGTAGT |
| *GFP-mut3-rev* | ACTGACTAGTTTATTTGTATAGTTCATCCATGCC |
| *IbAp-fwd* | GAGCTTAATTAAAATTCATCTGTTGATCGTGG |
| *IbAp-rev* | GATATCTAGATAGCTCCTGAAATCAGCGAGAATGTAAG |
| *mCherry-fwd* | TCACCACCATCATTAGGATGGTGGTGATGATAATGGTTAGCAAAGGTGAAGAGGA |
| *mCherry-rev* | GGTGCTCGAGTGCGGCCGCAAGCTTTCATTTATACAGTTCATCCA |
| *BRCA1-BRCT-fwd* | ACTTTAAGAAGGAGATATACATATGGTCAACAAAAGAATGTCCATGGTG |
| *BRCA1-BRCT-rev2* | CACCATCCTAATGATGGTGGTGATGATGACTAGTCACAGGTGCCTCACACATCT |
| *PARP1-BRCT-fwd* | ACTTTAAGAAGGAGATATACATATGGTGAACTCCTCTGCT |
| *PARP1-BRCT-rev* | CACCATCCTAATGATGGTGGTGATGATGACTAGTTGGGGCCACAACTTCAACA |
| *E6-fwd* | ACTTTAAGAAGGAGATATACATATGGC |
| *P19-fwd* | ACTTTAAGAAGGAGATATACATATGCTG |
| *Coupling cassette-rev* | CACCATCCTAATGATGGTGGTGA |
| *NusA-fwd* | CGACATATGAACAAAGAAATTTTGGCTGTA GT |
| *NusA-rev* | ATACATATGGCTACCAGAGCCGCTACCCGCTTCGTCACCGAACCAG |
| *SUMO-fwd* | GACCATATGTCGGACTCAGAAGTCAAT |
| *SUMO-rev* | ATCCATATGGCTACCAGAGCCGCTACCACCACCAATCTGTTCTCTGT |
| *Mut-PARP1-BRCT-fwd* | GAGATATACATATGGTGAACTCCTCTGCT |
| *Mutagenesis-fwd* | CCCCTCTAGAAATAATTTTGTTTAACTTTAAGAAGGAGATATACATATG |
| *Mutagenesis-rev* | CCTAATGATGGTGGTGATGATGACTAGT |
| *Library-seq-fwd* | TCGTCGGCAGCGTCAGATGTGTATAAGAGACAGAACTTTAAGAAGGAGATATACATATG |
| *PARP1-BRCT-int-rev* | GTCTCGTGGGCTCGGAGATGTGTATAAGAGACAGTGAAGGCTCTTGGTGGAG |
| *PARP1-BRCT-int-fwd* | TCGTCGGCAGCGTCAGATGTGTATAAGAGACAGAACAAGGATGAAGTGAAGGC |
| *Library-seq-rev* | GTCTCGTGGGCTCGGAGATGTGTATAAGAGACAGATGATGGTGGTGATGATGACTA |
| *CI2-fwd* | ACTTTAAGAAGGAGATATACATATGAAGACAGAGTGGCCAGAGTTGGTGGGG |
| *CI2-rev* | CACCATCCTAATGATGGTGGTGATGATGGCCGACCCTGGGGACCTGGGCAATG |
| *CI2-V34G-fwd* | ATCATAGTTCTGCCGGGGGGGACAATTGTGACC |
| *CI2-V34G-rev* | GGTCACAATTGTCCCCCCCGGCAGAACTATGAT |
| *CI2-R48I-fwd* | ATATCGGATCGACCGCGTCATACTCTTTGTCGATAAACTC |
| *CI2-R48I-rev* | GAGTTTATCGACAAAGAGTATGACGCGGTCGATCCGATAT |
| *CI2-I57A-fwd* | CCTGGGGACCTGGGCAGCGTTGTCGAGTTTATCG |
| *CI2-I57A-rev* | CGATAAACTCGACAACGCTGCCCAGGTCCCCAGG |
| *CI2-F50A-fwd* | GACCGCGTCCGCCTCGCGGTCGATAAACTCGA |
| *CI2-F50A-rev* | TCGAGTTTATCGACCGCGAGGCGGACGCGGTC |
| *CI2-V63A-fwd* | CAGGTCCCCAGGGCCGGCCATCATCAC |
| *CI2-V63A-rev* | GTGATGATGGCCGGCCCTGGGGACCTG |

**Supplementary Table 2. DNA and protein sequences for construction of vector and experimentally tested constructs**

| *Name* | *DNA sequence* | *Protein sequence* |
| --- | --- | --- |
| *lbpAp* | AATTCATCTGTTGATCGTGGGTGTTGGCCTGATGAGTTATAGCGATCCCTTGCTGAAAATAACATCATCATTACGTCGCACTGTGGCGGCTATCGCACTTTAACGTTTCGTGCTGCCCCCTCAGTCTATGCAATAGACCATAAACTGCAAAAAAAAGTCCGCTGATAAGGCTTGAAAAGTTCATTTCCAGACCCATTTTTACATCGTAGCCGATGAGGACGCGCCTGATGGGTGTTCTGGCTACCTGACCTGTCCATTGTGGAAGGTCTTACATTCTCGCTGATTTCAGGAGCTA |  |
| *GFP-ASV* | ATGCGTAAAGGAGAAGAACTTTTCACTGGAGTTGTCCCAATTCTTGTTGAATTAGATGGTGATGTTAATGGGCACAAATTTTCTGTCAGTGGAGAGGGTGAAGGTGATGCAACATACGGAAAACTTACCCTTAAATTTATTTGCACTACTGGAAAACTACCTGTTCCATGGCCAACACTTGTCACTACTTTCGGTTATGGTGTTCAATGCTTTGCGAGATACCCAGATCATATGAAACAGCATGACTTTTTCAAGAGTGCCATGCCCGAAGGTTATGTACAGGAAAGAACTATATTTTTCAAAGATGACGGGAACTACAAGACACGTGCTGAAGTCAAGTTTGAAGGTGATACCCTTGTTAATAGAATCGAGTTAAAAGGTATTGATTTTAAAGAAGATGGAAACATTCTTGGACACAAATTGGAATACAACTATAACTCACACAATGTATACATCATGGCAGACAAACAAAAGAATGGAATCAAAGTTAACTTCAAAATTAGACACAACATTGAAGATGGAAGCGTTCAACTAGCAGACCATTATCAACAAAATACTCCAATTGGCGATGGCCCTGTCCTTTTACCAGACAACCATTACCTGTCCACACAATCTGCCCTTTCGAAAGATCCCAACGAAAAGAGAGACCACATGGTCCTTCTTGAGTTTGTAACAGCTGCTGGGATTACACATGGCATGGATGAACTATACAAAAGGCCTGCAGCAAACGACGAAAACTACGCTGCATCAGTTTAA | MRKGEELFTGVVPILVELDGDVNGHKFSVSGEGEGDATYGKLTLKFICTTGKLPVPWPTLVTTFGYGVQCFARYPDHMKQHDFFKSAMPEGYVQERTIFFKDDGNYKTRAEVKFEGDTLVNRIELKGIDFKEDGNILGHKLEYNYNSHNVYIMADKQKNGIKVNFKIRHNIEDGSVQLADHYQQNTPIGDGPVLLPDNHYLSTQSALSKDPNEKRDHMVLLEFVTAAGITHGMDELYKRPAANDENYAASV |
| *GFP-mut3* | ATGCGTAAAGGAGAAGAACTTTTCACTGGAGTTGTCCCAATTCTTGTTGAATTAGATGGTGATGTTAATGGGCACAAATTTTCTGTCAGTGGAGAGGGTGAAGGTGATGCAACATACGGAAAACTTACCCTTAAATTTATTTGCACTACTGGAAAACTACCTGTTCCATGGCCAACACTTGTCACTACTTTCGGTTATGGTGTTCAATGCTTTGCGAGATACCCAGATCATATGAAACAGCATGACTTTTTCAAGAGTGCCATGCCCGAAGGTTATGTACAGGAAAGAACTATATTTTTCAAAGATGACGGGAACTACAAGACACGTGCTGAAGTCAAGTTTGAAGGTGATACCCTTGTTAATAGAATCGAGTTAAAAGGTATTGATTTTAAAGAAGATGGAAACATTCTTGGACACAAATTGGAATACAACTATAACTCACACAATGTATACATCATGGCAGACAAACAAAAGAATGGAATCAAAGTTAACTTCAAAATTAGACACAACATTGAAGATGGAAGCGTTCAACTAGCAGACCATTATCAACAAAATACTCCAATTGGCGATGGCCCTGTCCTTTTACCAGACAACCATTACCTGTCCACACAATCTGCCCTTTCGAAAGATCCCAACGAAAAGAGAGACCACATGGTCCTTCTTGAGTTTGTAACAGCTGCTGGGATTACACATGGCATGGATGAACTATACAAATAA | MRKGEELFTGVVPILVELDGDVNGHKFSVSGEGEGDATYGKLTLKFICTTGKLPVPWPTLVTTFGYGVQCFARYPDHMKQHDFFKSAMPEGYVQERTIFFKDDGNYKTRAEVKFEGDTLVNRIELKGIDFKEDGNILGHKLEYNYNSHNVYIMADKQKNGIKVNFKIRHNIEDGSVQLADHYQQNTPIGDGPVLLPDNHYLSTQSALSKDPNEKRDHMVLLEFVTAAGITHGMDELYK |
| *Translation coupling cassette* | ACTAGTCATCATCACCACCATCATTAGGATGGTGGTGATGATA |  |
| *mCherry* | ATGGTTAGCAAAGGTGAAGAGGATAATATGGCCATCATCAAAGAATTTATGCGCTTTAAAGTGCACATGGAAGGTAGCGTTAATGGCCATGAATTTGAAATTGAAGGTGAAGGCGAAGGTCGTCCGTATGAAGGCACCCAGACCGCAAAACTGAAAGTTACCAAAGGTGGTCCGCTGCCGTTTGCATGGGATATTCTGAGTCCGCAGTTTATGTATGGTAGCAAAGCCTATGTTAAACATCCGGCAGATATCCCGGATTATCTGAAACTGAGCTTTCCGGAAGGTTTTAAATGGGAACGTGTGATGAATTTTGAAGATGGTGGTGTGGTGACCGTTACCCAGGATAGCAGCCTGCAGGATGGTGAATTTATCTATAAAGTTAAACTGCGTGGCACCAATTTTCCGAGTGATGGTCCGGTTATGCAGAAAAAAACAATGGGTTGGGAAGCAAGCAGCGAACGTATGTATCCGGAAGATGGCGCACTGAAAGGTGAAATTAAACAGCGCCTGAAACTGAAAGATGGTGGCCATTATGATGCAGAAGTTAAAACCACCTATAAAGCCAAAAAACCGGTTCAGCTGCCTGGTGCATATAACGTTAACATTAAACTGGATATCACCAGCCACAACGAGGATTATACCATTGTTGAACAGTATGAACGTGCAGAAGGTCGCCATAGTACCGGTGGTATGGATGAACTGTATAAATGA | MVSKGEEDNMAIIKEFMRFKVHMEGSVNGHEFEIEGEGEGRPYEGTQTAKLKVTKGGPLPFAWDILSPQFMYGSKAYVKHPADIPDYLKLSFPEGFKWERVMNFEDGGVVTVTQDSSLQDGEFIYKVKLRGTNFPSDGPVMQKKTMGWEASSERMYPEDGALKGEIKQRLKLKDGGHYDAEVKTTYKAKKPVQLPGAYNVNIKLDITSHNEDYTIVEQYERAEGRHSTGGMDELYK |
| *PARP1-BRCT* | GTGAACTCCTCTGCTTCAGCAGATAAGCCATTATCCAACATGAAGATCCTGACTCTCGGGAAGCTGTCCCGGAACAAGGATGAAGTGAAGGCCATGATTGAGAAACTCGGGGGGAAGTTGACGGGGACGGCCAACAAGGCTTCCCTGTGCATCAGCACCAAAAAGGAGGTGGAAAAGATGAATAAGAAGATGGAGGAAGTAAAGGAAGCCAACATCCGAGTTGTGTCTGAGGACTTCCTCCAGGACGTCTCCGCCTCCACCAAGAGCCTTCAGGAGTTGTTCTTAGCGCACATCTTGTCCCCTTGGGGGGCAGAGGTGAAGGCAGAGCCTGTTGAAGTTGTGGCCCC | VNSSASADKPLSNMKILTLGKLSRNKDEVKAMIEKLGGKLTGTANKASLCISTKKEVEKMNKKMEEVKEANIRVVSEDFLQDVSASTKSLQELFLAHILSPWGAEVKAEPVEVVAP |
| *BRCA1-BRCT-truncated* | GTCAACAAAAGAATGTCCATGGTGGTGTCTGGCCTGACCCCAGAAGAATTTATGCTCGTGTACAAGTTTGCCAGAAAACACCACATCACTTTAACTAATCTAATTACTGAAGAGACTACTCATGTTGTTATGAAAACAGATGCTGAGTTTGTGTGTGAACGGACACTGAAATATTTTCTAGGAATTGCGGGAGGAAAATGGGTAGTTAGCTATTTCTGGGTGACCCAGTCTATTAAAGAAAGAAAAATGCTGAATGAGCATGATTTTGAAGTCAGAGGAGATGTGGTCAATGGAAGAAACCACCAAGGTCCAAAGCGAGCAAGAGAATCCCAGGACAGAAAGATCTTCAGGGGGCTAGAAATCTGTTGCTATGGGCCCTTCACCAACATGCCCACAGATCAACTGGAATGGATGGTACAGCTGTGTGGTGCTTCTGTGGTGAAGGAGCTTTCATCATTCACCCTTGGCACAGGTGTCCACCCAATTGTGGTTGTGCAGCCAGATGCCTGGACAGAGGACAATGGCTTCCATGCAATTGGGCAGATGTGTGAGGCACCTGTG | VNKRMSMVVSGLTPEEFMLVYKFARKHHITLTNLITEETTHVVMKTDAEFVCERTLKYFLGIAGGKWVVSYFWVTQSIKERKMLNEHDFEVRGDVVNGRNHQGPKRARESQDRKIFRGLEICCYGPFTNMPTDQLEWMVQLCGASVVKELSSFTLGTGVHPIVVVQPDAWTEDNGFHAIGQMCEAPV |
| *P19* | CTGCTGGAAGAAGTTCGCGCAGGCGATCGTCTGAGCGGTGCAGCAGCACGTGGTGATGTTCAAGAAGTGCGTCGTCTGCTGCATCGTGAACTGGTTCATCCTGATGCACTGAATCGTTTTGGTAAAACCGCACTGCAGGTTATGATGTTTGGTAGCACCGCAATTGCACTGGAACTGCTGAAACAGGGTGCAAGCCCGAATGTTCAGGATACCAGCGGCACCAGTCCGGTTCATGATGCCGCACGTACCGGTTTTCTGGATACCCTGAAAGTTCTGGTTGAACATGGTGCAGATGTTAATGTTCCGGATGGTACAGGTGCACTGCCGATTCATCTGGCCGTGCAAGAAGGTCATACCGCAGTTGTTAGCTTTCTGGCAGCAGAAAGCGATCTGCATCGTCGTGATGCACGTGGTCTGACACCGCTGGAACTGGCACTGCAGCGTGGTGCACAGGATCTGGTTGATATTCTGCAGGGTCACATGGTTGCACCGCTG | LLEEVRAGDRLSGAAARGDVQEVRRLLHRELVHPDALNRFGKTALQVMMFGSTAIALELLKQGASPNVQDTSGTSPVHDAARTGFLDTLKVLVEHGADVNVPDGTGALPIHLAVQEGHTAVVSFLAAESDLHRRDARGLTPLELALQRGAQDLVDILQGHMVAPL |
| *E6* | GCGCGCTTTGAGGATCCAACACGGCGACCCTACAAGCTACCTGATCTGTGCACGGAACTGAACACTTCACTGCAAGACATAGAAATAACCTGTGTATATTGCAAGACAGTATTGGAACTTACAGAGGTATTTGAATTTGCATTTAAAGATTTATTTGTGGTGTATAGAGACAGTATACCGCATGCTGCATGCCATAAATGTATAGATTTTTATTCTAGAATTAGAGAATTAAGACATTATTCAGACTCTGTGTATGGAGACACATTGGAAAAACTAACTAACACTGGGTTATACAATTTATTAATAAGGTGCCTGCGGTGCCAGAAACCGTTGAATCCAGCAGAAAAACTTAGACACCTTAATGAAAAACGACGATTCCACAACATAGCTGGGCACTATAGAGGCCAGTGCCATTCGTGCTGCAACCGAGCACGACAGGAAAGACTCCAACGACGCAGAGAAACACAAGTA | ARFEDPTRRPYKLPDLCTELNTSLQDIEITCVYCKTVLELTEVFEFAFKDLFVVYRDSIPHAACHKCIDFYSRIRELRHYSDSVYGDTLEKLTNTGLYNLLIRCLRCQKPLNPAEKLRHLNEKRRFHNIAGHYRGQCHSCCNRARQERLQRRRETQV |
| *NusA* | AACAAAGAAATTTTGGCTGTAGTTGAAGCCGTATCCAATGAAAAGGCGCTACCTCGCGAGAAGATTTTCGAAGCATTGGAAAGCGCGCTGGCGACAGCAACAAAGAAAAAATATGAACAAGAGATCGACGTCCGCGTACAGATCGATCGCAAAAGCGGTGATTTTGACACTTTCCGTCGCTGGTTAGTTGTTGATGAAGTCACCCAGCCGACCAAGGAAATCACCCTTGAAGCCGCACGTTATGAAGATGAAAGCCTGAACCTGGGCGATTACGTTGAAGATCAGATTGAGTCTGTTACCTTTGACCGTATCACTACCCAGACGGCAAAACAGGTTATCGTGCAGAAAGTGCGTGAAGCCGAACGTGCGATGGTGGTTGATCAGTTCCGTGAACACGAAGGTGAAATCATCACCGGCGTGGTGAAAAAAGTAAACCGCGACAACATCTCTCTGGATCTGGGCAACAACGCTGAAGCCGTGATCCTGCGCGAAGATATGCTGCCGCGTGAAAACTTCCGCCCTGGCGACCGCGTTCGTGGCGTGCTCTATTCCGTTCGCCCGGAAGCGCGTGGCGCGCAACTGTTCGTCACTCGTTCCAAGCCGGAAATGCTGATCGAACTGTTCCGTATTGAAGTGCCAGAAATCGGCGAAGAAGTGATTGAAATTAAAGCAGCGGCTCGCGATCCGGGTTCTCGTGCGAAAATCGCGGTGAAAACCAACGATAAACGTATCGATCCGGTAGGTGCTTGCGTAGGTATGCGTGGCGCGCGTGTTCAGGCGGTGTCTACTGAACTGGGTGGCGAGCGTATCGATATCGTCCTGTGGGATGATAACCCGGCGCAGTTCGTGATTAACGCAATGGCACCGGCAGACGTTGCTTCTATCGTGGTGGATGAAGATAAACACACCATGGATATCGCCGTTGAAGCCGGTAACCTGGCGCAGGCGATTGGCCGTAACGGTCAGAACGTGCGTCTGGCTTCGCAGCTGAGCGGTTGGGAACTCAACGTGATGACCGTTGACGACCTGCAGGCTAAGCATCAGGCGGAAGCGCACGCAGCGATCGACACCTTCACCAAATATCTCGACATCGACGAAGACTTCGCGACTGTTCTGGTAGAAGAAGGCTTCTCGACGCTGGAAGAATTGGCCTATGTGCCGATGAAAGAGCTGTTGGAAATCGAAGGCCTTGATGAGCCGACCGTTGAAGCACTGCGCGAGCGTGCTAAAAATGCACTGGCCACCATTGCACAGGCCCAGGAAGAAAGCCTCGGTGATAACAAACCGGCTGACGATCTGCTGAACCTTGAAGGGGTAGATCGTGATTTGGCATTCAAACTGGCCGCCCGTGGCGTTTGTACGCTGGAAGATCTCGCCGAACAGGGCATTGATGATCTGGCTGATATCGAAGGGTTGACCGACGAAAAAGCCGGAGCACTGATTATGGCTGCCCGTAATATTTGCTGGTTCGGTGACGAAGCGGGTAGCGGCTCTGGTAGC | NKEILAVVEAVSNEKALPREKIFEALESALATATKKKYEQEIDVRVQIDRKSGDFDTFRRWLVVDEVTQPTKEITLEAARYEDESLNLGDYVEDQIESVTFDRITTQTAKQVIVQKVREAERAMVVDQFREHEGEIITGVVKKVNRDNISLDLGNNAEAVILREDMLPRENFRPGDRVRGVLYSVRPEARGAQLFVTRSKPEMLIELFRIEVPEIGEEVIEIKAAARDPGSRAKIAVKTNDKRIDPVGACVGMRGARVQAVSTELGGERIDIVLWDDNPAQFVINAMAPADVASIVVDEDKHTMDIAVEAGNLAQAIGRNGQNVRLASQLSGWELNVMTVDDLQAKHQAEAHAAIDTFTKYLDIDEDFATVLVEEGFSTLEELAYVPMKELLEIEGLDEPTVEALRERAKNALATIAQAQEESLGDNKPADDLLNLEGVDRDLAFKLAARGVCTLEDLAEQGIDDLADIEGLTDEKAGALIMAARNICWFGDEAGSGSGS |
| *SUMO* | TCGGACTCAGAAGTCAATCAAGAAGCTAAGCCAGAGGTCAAGCCAGAAGTCAAGCCTGAGACTCACATCAATTTAAAGGTGTCCGATGGATCTTCAGAGATCTTCTTCAAGATCAAAAAGACCACTCCTTTAAGAAGGCTGATGGAAGCGTTCGCTAAAAGACAGGGTAAGGAAATGGACTCCTTAAGATTCTTGTACGACGGTATTAGAATTCAAGCTGATCAGACCCCTGAAGATTTGGACATGGAGGATAACGATATTATTGAGGCTCACAGAGAACAGATTGGTGGTGGTAGCGGCTCTGGTAGC | SDSEVNQEAKPEVKPEVKPETHINLKVSDGSSEIFFKIKKTTPLRRLMEAFAKRQGKEMDSLRFLYDGIRIQADQTPEDLDMEDNDIIEAHREQIGGGSGSGS |
| CI2 | ATGAAGACAGAGTGGCCAGAGTTGGTGGGGAAATCGGTGGAGGAGGCCAAGAAGGTGATTCTGCAGGACAAGCCAGAGGCGCAAATCATAGTTCTGCCGGTGGGGACAATTGTGACCATGGAATATCGGATCGACCGCGTCCGCCTCTTTGTCGATAAACTCGACAACATTGCCCAGGTCCCCAGGGTCGGC | MKTEWPELVGKSVEEAKKVILQDKPEAQIIVLPVGTIVTMEYRIDRVRLFVDKLDNIAQVPRVG |

Supplementary Table 3. Primers for construction of PARP1-BRCT mutants

| **Mutant** | **Fragment 1** | **Fragment 2** |
| --- | --- | --- |
| **G20V** | G20V-fwd (ctgactctcGTgaagctgtccc)  +  pET22-mut-rev (ctcacgctgtaggtatctcagttcg) | G20V- Rev (GGGACAGCTTCACGAGAGTCAG)  +  pET22-mut-fwd (cgaactgagatacctacagcgtgag) |
| **G20W** | G20W-fwd (atcctgactctcTggaagctgtccc)  pET22-mut-rev (ctcacgctgtaggtatctcagttcg) | G20W- Rev (GGGACAGCTTCCAGAGAGTCAGGAT)  +  pET22-mut-fwd (cgaactgagatacctacagcgtgag) |
| **A31T** | A31T-fwd (GAtgaagtgaagAccatgaTtgag)  +  pET22-mut-rev (ctcacgctgtaggtatctcagttcg) | A31T-Rev (CTCAATCATGGTCTTCACTTCATC)  +  pET22-mut-fwd (cgaactgagatacctacagcgtgag) |
| **I33N** | I33N-FWD (gaaggccatgaAtgagaaactcg)  +  pET22-mut-rev (ctcacgctgtaggtatctcagttcg) | I33N-REV (CGAGTTTCTCATTCATGGCCTTC)  +  pET22-mut-fwd (cgaactgagatacctacagcgtgag) |
| **I33T** | I33T-fwd (gaaggccatgaCtgagaaactcg)  +  pET22-mut-rev (ctcacgctgtaggtatctcagttcg) | I33T-REV (CGAGTTTCTCAGTCATGGCCTTC)  +  pET22-mut-fwd (cgaactgagatacctacagcgtgag) |
| **G37E** | G37E-fwd (tgagaaactcgAggggaagttga)  +  pET22-mut-rev (ctcacgctgtaggtatctcagttcg) | G37E- Rev (TCAACTTCCCCTCGAGTTTCTCA)  +  pET22-mut-fwd (cgaactgagatacctacagcgtgag) |
| **G37R** | G37R-fwd (tgagaaactcCGgggGAagttga)  +  pET22-mut-rev (ctcacgctgtaggtatctcagttcg) | G37R- Rev (TCAACTTCCCCCGGAGTTTCTCA)  +  pET22-mut-fwd (cgaactgagatacctacagcgtgag) |
| **G37W** | G37W-fwd (tgagaaactcTGGggGaAgttga)  +  pET22-mut-rev (ctcacgctgtaggtatctcagttcg) | G37W- Rev (TCAACTTCCCCCAGAGTTTCTCA)  +  pET22-mut-fwd (cgaactgagatacctacagcgtgag) |
| **G38R** | G38R-fwd (agaaactcGGGCgGaAgttgaC)  +  pET22-mut-rev (ctcacgctgtaggtatctcagttcg) | G38R-Rev (GTCAACTTCCGCCCGAGTTTCT)  +  pET22-mut-fwd (cgaactgagatacctacagcgtgag) |
| **C50Y** | C50Y-Fwd (GCTTCCCTGTACATCAGCACCAA)  +  pET22-mut-rev (ctcacgctgtaggtatctcagttcg) | C50Y-ReV (TTGGTGCTGATGTACAGGGAAGC)  +  pET22-mut-fwd (cgaactgagatacctacagcgtgag) |
| **S52I** | S52I-fwd (cctgtgcatcaTcaccaaaaag)  +  pET22-mut-rev (ctcacgctgtaggtatctcagttcg) | S52I- Rev (CTTTTTGGTGATGATGCACAGG)  +  pET22-mut-fwd (cgaactgagatacctacagcgtgag) |
| **S52N** | S52N-fwd (ctgtgcatcAAcaccaaaaagGAG)  +  pET22-mut-rev (ctcacgctgtaggtatctcagttcg) | S52N- Rev (CTCCTTTTTGGTGTTGATGCACAG)  +  pET22-mut-fwd (cgaactgagatacctacagcgtgag) |
| **I72N** | I72N-fwd (ggaagccaacaAccgagttgtg)  +  pET22-mut-rev (ctcacgctgtaggtatctcagttcg) | I72N- Rev (CACAACTCGGTTGTTGGCTTCC)  +  pET22-mut-fwd (cgaactgagatacctacagcgtgag) |
| **V74F** | V74F-fwd (ccaacatccgaTttgtgtctgag)  +  pET22-mut-rev (ctcacgctgtaggtatctcagttcg) | V74F- Rev (CTCAGACACAAATCGGATGTTGG)  +  pET22-mut-fwd (cgaactgagatacctacagcgtgag) |
| **V74I** | V74I-fwd (ccaacatccgaAttgtgtctgag)  +  pET22-mut-rev (ctcacgctgtaggtatctcagttcg) | V74I- Rev (CTCAGACACAATTCGGATGTTGG)  +  pET22-mut-fwd (cgaactgagatacctacagcgtgag) |
| **D78V** | D78V-fwd (tgtgtctgaggTcttcctccagg)  +  pET22-mut-rev (ctcacgctgtaggtatctcagttcg) | D78V- Rev (CCTGGAGGAAGACCTCAGACACA)  +  pET22-mut-fwd (cgaactgagatacctacagcgtgag) |
| **Q81R** | Q81R -fwd (gacttcctccGggacgtctcc)  +  pET22-mut-rev (ctcacgctgtaggtatctcagttcg) | Q81R – Rev (GGAGACGTCCCGGAGGAAGTC)  +  pET22-mut-fwd (cgaactgagatacctacagcgtgag) |
| **A96P** | A96P-fwd (gttgttcttaCcgcacatcttgtc)  +  pET22-mut-rev (ctcacgctgtaggtatctcagttcg) | A96P- Rev (GGACAAGATGTGCGGTAAGAACAAC)  +  pET22-mut-fwd (cgaactgagatacctacagcgtgag) |
| **A96V** | A96V-fwd (gagttgttcttagTgcacatcttgtcc)  +  pET22-mut-rev (ctcacgctgtaggtatctcagttcg) | A96V- Rev (GGACAAGATGTGCACTAAGAACAACTC)  +  pET22-mut-fwd (cgaactgagatacctacagcgtgag) |
| **H97L** | H97L-fwd (gttcttaGcgcTcatcttgtccc)  +  pET22-mut-rev (ctcacgctgtaggtatctcagttcg) | H97L- Rev (GGGACAAGATGAGCGCTAAGAAC)  +  pET22-mut-fwd (cgaactgagatacctacagcgtgag) |
