# Supplementary material for: A dual-reporter system for investigating and optimizing protein translation and folding in E. coli
Source: Nat Commun. 2021 Oct 19;12:6093. doi: 10.1038/s41467-021-26337-1 (PMC8526717; doi:10.1038/s41467-021-26337-1)
Supplement: Supplementary file 2 — Reporting Summary [file 41467_2021_26337_MOESM2_ESM.pdf]

nature research

Corresponding author(s): Alex Toftgaard Nielsen

Last updated by author(s): Sep 23, 2021

Reporting Summary

Nature Research wishes to improve the reproducibility of the work that we publish. This form provides structure for consistency and transparency in reporting. For further information on Nature Research policies, see Authors & Referees and the Editorial Policy Checklist .

Statistics

For all statistical analyses, confirm that the following items are present in the figure legend, table legend, main text, or Methods section.

n/a	Confirmed	
☐	☒	The exact sample size (*n*) for each experimental group/condition, given as a discrete number and unit of measurement
☐	☒	A statement on whether measurements were taken from distinct samples or whether the same sample was measured repeatedly
☒	☐	The statistical test(s) used AND whether they are one- or two-sided<br>*Only common tests should be described solely by name; describe more complex techniques in the Methods section.*
☒	☐	A description of all covariates tested
☒	☐	A description of any assumptions or corrections, such as tests of normality and adjustment for multiple comparisons
☐	☒	A full description of the statistical parameters including central tendency (e.g. means) or other basic estimates (e.g. regression coefficient) AND variation (e.g. standard deviation) or associated estimates of uncertainty (e.g. confidence intervals)
☒	☐	For null hypothesis testing, the test statistic (e.g. *F*, *t*, *r*) with confidence intervals, effect sizes, degrees of freedom and *P* value noted<br>*Give P values as exact values whenever suitable.*
☒	☐	For Bayesian analysis, information on the choice of priors and Markov chain Monte Carlo settings
☒	☐	For hierarchical and complex designs, identification of the appropriate level for tests and full reporting of outcomes
☒	☐	Estimates of effect sizes (e.g. Cohen's *d*, Pearson's *r*), indicating how they were calculated

Our web collection on statistics for biologists contains articles on many of the points above.

Software and code

Policy information about availability of computer code

Data collection	FACS data was collected using the BD FACS-Diva software
Data analysis	- The Enrich analysis was carried out using Enrich2 software (reference 36). Fastq files were converted into Enrich2 compatible variant counts using python scripts. The scripts for doing this as well as an Enrich2 analysis config file are available at https://doi.org/10.11583/DTU.10265420.<br>- Decoy structures were generated using Rosetta's threading protocol (reference 38).<br>- Western Blot signals were quantified using the public Image J software (reference 52).<br>- In silico calculations of thermodynamic stability (DDG) was carried out using FoldX (reference 37)<br>- Data was analyzed using Microsoft Office Excel for mac (version 16.16.17)<br>- FACS data was analyzed using FlowJo V10

For manuscripts utilizing custom algorithms or software that are central to the research but not yet described in published literature, software must be made available to editors/reviewers. We strongly encourage code deposition in a community repository (e.g. GitHub). See the Nature Research guidelines for submitting code & software for further information.

Data

Policy information about availability of data

All manuscripts must include a data availability statement. This statement should provide the following information, where applicable:

- Accession codes, unique identifiers, or web links for publicly available datasets
- A list of figures that have associated raw data
- A description of any restrictions on data availability

The data availability statement in the manuscript reads: "Source data are provided with this paper in the supplementary document named "Source data". Additional data can be found at https://doi.org/10.11583/DTU.c.5633536.v164. Biological materials are available from the corresponding author upon request."

Field-specific reporting

Please select the one below that is the best fit for your research. If you are not sure, read the appropriate sections before making your selection.

- [x] Life sciences
- [ ] Behavioural & social sciences
- [ ] Ecological, evolutionary & environmental sciences

For a reference copy of the document with all sections, see nature.com/documents/nr-reporting-summary-flat.pdf

Life sciences study design

All studies must disclose on these points even when the disclosure is negative.

Sample size	No sample size calculations were performed. For identifying mutations that destabilize PARP1-BRCT, a library of more than 100.000 random mutants was generated. Given a average mutation rate of 1 nucleotide per gene (350 nucleotides) the library size was estimated to be sufficient. Less that 5% (corresponding approximately 5000 cells in the original library) showed GFP fluorescence matching that of a misfolded protein. Of these, a total of 150.000 cells were collected in two rounds of sorting. We therefore estimate that the sample size was sufficient.
Data exclusions	No data was excluded from the analysis
Replication	Experiments were carried out at least in triplicates when relevant and all replicates were successful and gave similar data as reported in the figures.
Randomization	Not relevant as no experimental groups were included in the study
Blinding	Group allocations were not included in the study and blinding was therefore not carried out

Reporting for specific materials, systems and methods

We require information from authors about some types of materials, experimental systems and methods used in many studies. Here, indicate whether each material, system or method listed is relevant to your study. If you are not sure if a list item applies to your research, read the appropriate section before selecting a response.

Materials & experimental systems

n/a	Involved in the study	
[ ]	[x]	Antibodies
[x]	[ ]	Eukaryotic cell lines
[x]	[ ]	Palaeontology
[x]	[ ]	Animals and other organisms
[x]	[ ]	Human research participants
[x]	[ ]	Clinical data

Methods

n/a	Involved in the study	
[x]	[ ]	ChIP-seq
[ ]	[x]	Flow cytometry
[x]	[ ]	MRI-based neuroimaging

Antibodies

Antibodies used	An anti-His antibody (Novagen, 70796-3) was used for quantifying histidine tagged proteins
Validation	The commonly used antibody successfully labeled his-tagged proteins in western blots, and no further validation was carried out.

Flow Cytometry

Plots

Confirm that:

- [x] The axis labels state the marker and fluorochrome used (e.g. CD4-FITC).
- [x] The axis scales are clearly visible. Include numbers along axes only for bottom left plot of group (a 'group' is an analysis of identical markers).
- [x] All plots are contour plots with outliers or pseudocolor plots.
- [x] A numerical value for number of cells or percentage (with statistics) is provided.

Methodology

Sample preparation	The methods are described several places in the Materials and Methods section, for example: IB formation in E. coli was induced by performing a heat-shock for 10 min at 42°C. After heat shock, cells were grown for an additional 2.5 hours at 37°C and 300 rpm. Induction of the lbpAp promoter by IBs in single cells was monitored over time by changes of the GFP signal using flow cytometry (Instrument: BD FACS-Aria™SORP cell sorter; Laser 1: 488 nm: >50 mW, Filter: 505LP, 530/30-nm FITC, Laser 2: 561 nm: >50 mW; Filter: 600LP, 610/20-nm PE-Texas Red®). As control, the GFP signal in un-induced cells was monitored for each time point. The GFP (FITC-A, X-mean) values at each time point analysed using the FlowJo V10 software were normalized to the corresponding background GFP signal.
Instrument	BD FACS-Aria™SORP cell sorter
Software	Data was collected using the BD FACS-Diva software.<br>Data was analyzed using FlowJo V10
Cell population abundance	The bacteria were abundant in the sample and no significant noise was observed during the analysis of the samples. A total of 150.000 cells were collected. Re-analysis of the collected cells was carried out in order to validate successful sorting of the gated cells.
Gating strategy	The gates used for sorting are shown in Figure 5 and 7.

- [ ] Tick this box to confirm that a figure exemplifying the gating strategy is provided in the Supplementary Information.
